# Association between echocardiographic structural parameters and body weight in Wistar rats

**DOI:** 10.18632/oncotarget.15320

**Published:** 2017-02-13

**Authors:** Silvio A. Oliveira-Junior, Paula F. Martinez, William Y.C. Fan, Bruno T. Nakatani, Luana U. Pagan, Carlos R. Padovani, Antonio C. Cicogna, Marina P. Okoshi, Katashi Okoshi

**Affiliations:** ^1^ School of Physical Therapy, Federal University of Mato Grosso do Sul, Campo Grande, MS, Brazil; ^2^ Botucatu Medical School, Sao Paulo State University, UNESP, Botucatu, SP, Brazil; ^3^ Botucatu Biosciences Institute, Sao Paulo State University, UNESP, Botucatu, SP, Brazil

**Keywords:** development, physiological cardiac remodeling, rat, echocardiogram, cardiac structures

## Abstract

**Background:**

The association between echocardiographic structural parameters and body weight (BW) during rat development has been poorly addressed. We evaluated echocardiographic variables: left ventricular (LV) end-diastolic (LVDD) and end-systolic (LVSD) diameters, LV diastolic posterior wall thickness (PWT), left atrial diameter (LA), and aortic diameter (AO) in function of BW during development.

**Results/Materials and Methods:**

Male Wistar rats (*n* = 328, BW: 302–702 g) were retrospectively used to construct regression models and 95% confidence intervals relating to cardiac structural parameters and BW. Adjusted indexes were significant to all relationships; the regression model for predicting LVDD (R2 = 0.678; *p* < 0.001) and AO (R2 = 0.567; *p* < 0.001) had the highest prediction coefficients and LA function the lowest prediction coefficient (R2 = 0.274; *p* < 0.01). These relationships underwent validation by performing echocardiograms on additional rats (*n* = 43, BW: 300–600 g) and testing whether results were within confidence intervals of our regressions. Prediction models for AO and LA correctly allocated 38 (88.4%) and 39 rats (90.7%), respectively, within the 95% confidence intervals. Regression models for LVDD, LVSD, and PWT included 27 (62.7%), 30 (69.8%), and 19 (44.2%) animals, respectively, within the 95% confidence intervals.

**Conclusions:**

Increase in cardiac structures is associated with BW gain during rat growth. LA and AO can be correctly predicted using regression models; prediction of PWT and LV diameters is not accurate.

## INTRODUCTION

Animal models are highly relevant in evaluating cardiac remodeling under different situations of injury and in developing treatment strategies for alleviating heart disease in humans [1–3]. Although large mammalian species are considered more relevant for simulating human disease, rats are commonly used for economic reasons.

Transthoracic echocardiography is a recognized safe, reliable, and repeatable diagnostic method which has been extensively used to evaluate structural and functional cardiac parameters in rats [4]. Being noninvasive in nature, it can be used to perform longitudinal studies on cardiac remodeling pathophysiology and treatment. Baseline echocardiographic values for cardiac anatomy and function have been documented for normal adult and aged rats [5–7].

Cardiac structural parameters such as left ventricular (LV) diastolic diameter, LV mass, and left atrial diameter are often normalized to body weight to compensate for small changes in body mass [8, 9]. However, this does not take into account that the relationship between cardiac structures and body size is nonlinear [10, 11]. This normalization may therefore under or overcorrect for the impact of body size when evaluating rats with severe body weight change conditions such as undernutrition, obesity, or cardiac and cancer cachexia. To date, the relationship between cardiac structures and body weight during rat development has not been investigated. This study evaluates echocardiographic variables LV end-diastolic and end-systolic diameters, LV diastolic posterior wall thickness, left atrial diameter, and aortic diameter in function of body weight during normal development in Wistar rats. The high number of ongoing rat cardiac remodeling studies in our laboratory allowed us to perform this work using a large number of animals [12–14].

## RESULTS

### Retrospective study

**Figure 1:**
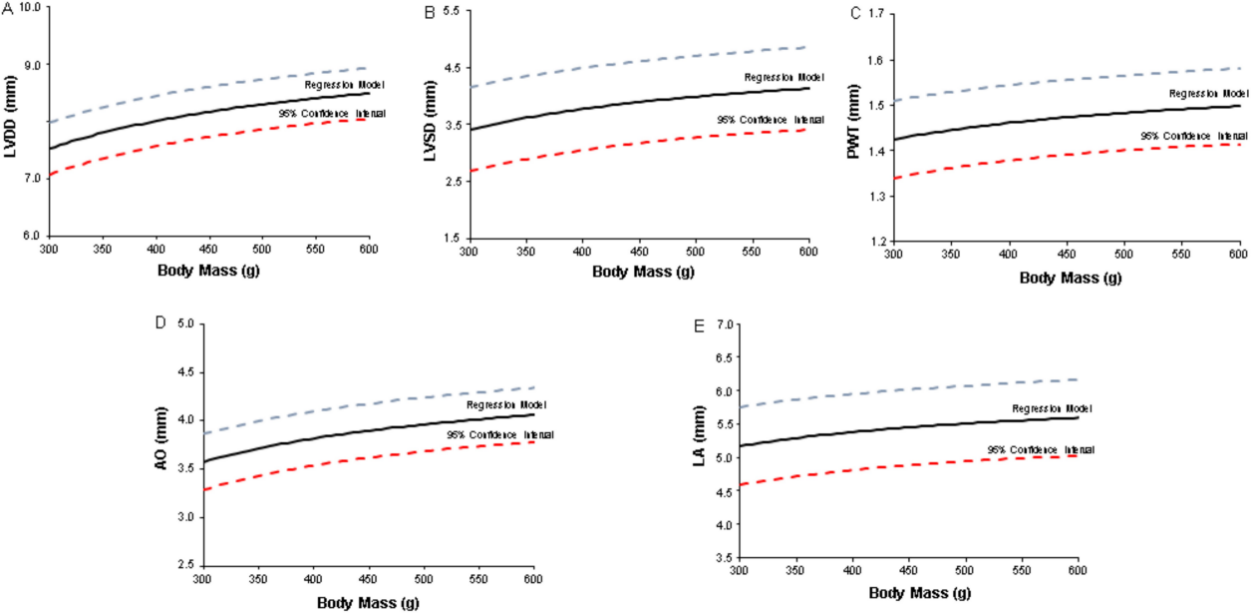
Regression models and 95% confidence intervals for (**A**) left ventricular (LV) end-diastolic diameter (LVDD), (**B**) LV end-systolic diameter (LVSD), (**C**) LV diastolic posterior wall thickness (PWT), (**D**) aortic diameter (AO), and (**E**) left atrial diameter (LA) in accordance with Wistar rat body weight. Straight line: regression model; dashed lines: superior and inferior 95% confidence intervals.

Rat body weight (*n* = 328) ranged from 302 to 702 g. Following descriptive analysis, 36 body mass categories were obtained (example: 300–309 g, 310–319 g, etc). Different regression models were then built to analyze the behavior of LVDD, LVSD, PWT, LA, and AO in function of the body weight (Table 1). Confidence intervals for each regression model are shown in Figure 1. Adjusted indexes were statistically significant for all relationships. The regression model for predicting LVDD and aortic diameter presented the highest prediction coefficients, and the model for predicting left atrial diameter the lowest prediction coefficient (Table 1).

**Table 1:** Non-linear regression models for cardiac structures in function of body weight

Regression	Coefficient of Determination (R^2^)	*P*-value	Variation Model	MSE
LVDD = 9.453–577.331/BW	0.678	*p* < 0.001	(LVDD)’ = 577.331/BW^2^	0.0451
LVSD = 4.845–428.215/BW	0.303	*p* < 0.001	(LVSD)’ = 428.215/BW^2^	0.1200
PWT = 1.571–43.730/BW	0.256	*p* < 0.005	(PWT)’ = 43.730/BW^2^	0.0016
AO = 4.542–289.199/BW	0.567	*p* < 0.001	(AO)’ = 289.199/BW^2^	0.0182
LA = 6.012–252.568/BW	0.274	*p* < 0.01	(LA)’ = 252.568/BW^2^	0.0749

LVDD: left ventricular (LV) end-diastolic diameter; LVSD: LV end-systolic diameter; PWT: LV posterior wall diastolic thickness; AO: aortic diameter; LA: left atrial diameter; BW: body weight; MSE: mean square error.

### Prospective study

**Figure 2:**
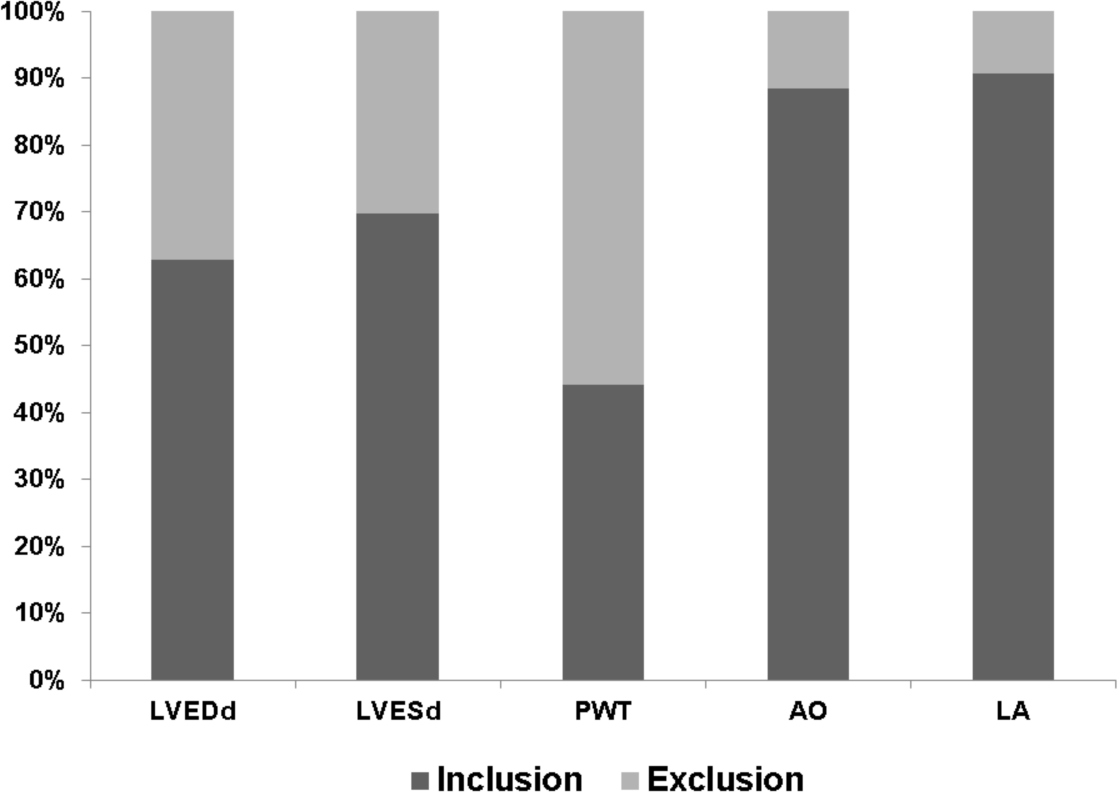
Percentages of echocardiographic parameters falling within regression model 95% confidence intervals for prospectively evaluated Wistar rats with body weights ranging from 300 to 600 g (*n* = 43) LVDD: left ventricular (LV) end-diastolic diameter, LVSD: LV end-systolic diameter, PWT: LV diastolic posterior wall thickness, AO: aortic diameter, LA: left atrial diameter.

To validate the relationships between cardiac structural parameters and body weight obtained in the retrospective study, we prospectively performed echocardiograms on additional rats (*n* = 43) with body weights ranging from 300 to 600 g and tested whether results were inside the confidence intervals of our regressions. Figure 2 shows parameter percentage values included in the 95% confidence intervals. Prediction models for aortic diameter and left atrial diameter correctly allocated 38 (88.4%) and 39 animals (90.7%), respectively, within the 95% confidence intervals. Regression models for LVDD and LVSD correctly included 27 (62.7%) and 30 (69.8%) rats, respectively, and regression function for PWT only correctly allocated 19 rats (44.2%).

## DISCUSSION

Transthoracic echocardiography has been extensively used to evaluate *in vivo* cardiac structures and ventricular function in rodents. This noninvasive technique allows longitudinal studies of cardiac remodeling induced by different types of aggression and the effects of therapeutic interventions [15–17]. Transthoracic echocardiography has also been used to examine cardiac remodeling caused by systemic diseases which are accompanied by losses or gains in body weight, such as undernutrition, cachexia, obesity, and aging [18–21]. In this context, cardiac structures are commonly related to body size to establish reference standards for normality and permit intergroup comparisons [22–24].

In this study, we aimed to construct 95% confidence intervals for normal cardiac structure values in rats with body weights ranging from 302 to 702 g. We selected echocardiographic parameters of cardiac structures that are classical descriptors in several cardiovascular conditions [25–27]. Despite linear relationships between cardiac structures and body mass in the early growth phase, these relationships become complex and nonlinear in later development periods up to adulthood. Therefore, as expected, all analyzed variables presented nonlinear relationships with body weight showing that during normal growth, cardiac structures do not increase linearly with body mass gain. To the best of our knowledge, this is the first study to evaluate associations between cardiac structures and body development up to adult age using a large sample of rats.

Although the regression model coefficient of determination for LVDD was strongly associated with body mass, prospective evaluation found that only 62.8% of LVDD values were within the 95% confidence intervals. Similarly, LVSD and PWT regression models demonstrated a poor potential for predicting values in normal rats (69.8% and 44.2%, respectively). However, despite presenting a low coefficient of determination in the retrospective study, the left atrial diameter regression model produced the best prospective prediction score (90.7%). The aortic diameter regression model was also a good predictor (88.4%) for normal rats. Therefore, normal values for left atrium and aorta diameters can be obtained by using our regression models when evaluating Wistar rats with different body weights. A similar approach was employed by Ahmet et al. [28] using sophisticated allometric scaling procedures to evaluate the effects of calorie restriction on cardioprotection in Fisher rats. As our LV diameter regression models included less than 70% of rats in the normal range, we should be careful when using mathematical models to estimate values for normal LV diameters.

A limitation of this study is that we evaluated only male Wistar rats. Therefore, additional studies are required to ascertain whether our results are valid for female Wistar rats and other rat strains.

In conclusion, cardiac structure increase is associated with body weight gain during Wistar rat growth. Left atrial and aortic diameters can be correctly predicted using regression models; however, left ventricular wall thickness and diameters prediction is not accurate.

## MATERIALS AND METHODS

### Animals

Two sets of animals were studied. The first set of rats was used to perform retrospective analysis and the second to prospectively validate results obtained in the initial study. All experiments and procedures were approved by the Ethics Committee of Botucatu Medical School, Sao Paulo State University, UNESP, SP, Brazil, and were in accordance with the “*Guide for the Care and Use of Laboratory Animals*” published by the US National Institutes of Health.

The retrospective study used data from male adult Wistar rats (*n* = 328) previously evaluated in our laboratory. All animals had been housed in a temperature controlled room at 23°C to 24°C on a 12-h light/dark cycle with free access to water and chow. Rats were euthanized at different ages and body weights according to the respective experimental protocols. Rats had been weighed and subjected to transthoracic echocardiography within one or two days of euthanasia.

For the prospective analysis, male Wistar rats (200–250 g, 50–60 days old; *n* = 43) were purchased from the Central Animal House at Botucatu Medical School, UNESP, and kept as previously described. These rats were assigned into seven groups (*n* = 6–7 per group) for body weight evaluation and echocardiogram after achieving the following body weights: 300, 350, 400, 450, 500, 550, and 600 g.

### Echocardiographic study

Echocardiographic evaluation was performed using a commercially available echocardiograph (General Electric Medical Systems, Vivid S6, Tirat Carmel, Israel) equipped with a 5–11.5 MHz multifrequency probe, as previously described [29–31]. Rats were anesthetized by intramuscular injection of ketamine (50 mg/kg) and xylazine (0.5 mg/kg). Two-dimensional guided M-mode images were obtained from parasternal short-axis views of the LV just below the tip of the mitral-valve leaflets, and at the level of the aortic valve and left atrium. M-mode images were printed on a thermal printer (Sony UP-890MD) at a sweep speed of 200 mm/s. All LV structures were manually measured by the same observer (KO) using the leading-edge method of the American Society of Echocardiography [32]. Mean values were obtained from at least five cardiac cycles on M-mode tracings. The following structural variables were measured: left atrial diameter, aortic diameter, LV end-diastolic and end-systolic diameters (LVDD and LVSD, respectively), and LV diastolic posterior wall thickness (PWT). Intraobserver reproducibility of echocardiographic variables has been previously published [4].

### Statistical analysis

All analyzes were carried out using the SPSS statistical software package (Release 6.0 for Windows; SPSS, Chicago, IL). In the retrospective study, relationships between body mass and echocardiographic parameters were determined from dispersion diagrams and regression models were constructed [33]. Higher coefficient of determination scores (*R2*) were considered for selecting the better prediction model. Using functions obtained by regression analysis, 95% confidence intervals were built and used to prospectively investigate the accuracy of predictive echocardiographic parameters values in healthy rats. Statistical significance was accepted at *P* < 0.05.

## Abbreviations

AO: aortic diameter
BW: body weight
LA: left atrial diameter
LV: left ventricular
LVDD: left ventricular end-diastolic diameter
LVSD: left ventricular end-systolic diameter
MSE: mean square error
PWT: left ventricular diastolic posterior wall thickness
